# A novel ADOA-associated *OPA1* mutation alters the mitochondrial function, membrane potential, ROS production and apoptosis

**DOI:** 10.1038/s41598-017-05571-y

**Published:** 2017-07-18

**Authors:** Juanjuan Zhang, Xiaoling Liu, Xiaoyang Liang, Yuanyuan Lu, Ling Zhu, Runing Fu, Yanchun Ji, Wenlu Fan, Jie Chen, Bing Lin, Yimin Yuan, Pingping Jiang, Xiangtian Zhou, Min-Xin Guan

**Affiliations:** 1 Division of Medical Genetics and Genomics, The Children’s Hospital, Zhejiang University School of Medicine, Hangzhou, Zhejiang 310058 China; 2 Institute of Genetics, Zhejiang University School of Medicine, Hangzhou, Zhejiang 310058 China; 3 School of Ophthalmology and Optometry, Wenzhou Medical University, Wenzhou, Zhejiang 325027 China; 4 Attardi Institute of Mitochondrial Biomedicine, Wenzhou Medical University, Wenzhou, Zhejiang 325035 China; 5 Collaborative Innovation Center for Diagnosis and Treatment of Infectious Diseases, Zhejiang University, Hangzhou, Zhejiang 310058 China

## Abstract

Autosomal dominant optic atrophy (ADOA) is a dominantly inherited optic neuropathy, affecting the specific loss of retinal ganglion cells (RGCs). The majority of affected cases of ADOA are associated with mutations in *OPA1* gene. Our previous investigation identified the c.1198C > G (p.P400A) mutation in the *OPA1* in a large Han Chinese family with ADOA. In this report, we performed a functional characterization using lymphoblostoid cell lines derived from affected members of this family and control subjects. Mutant cell lines exhibited the aberrant mitochondrial morphology. A ~24.6% decrease in the mitochondrial DNA (mtDNA) copy number was observed in mutant cell lines, as compared with controls. Western blotting analysis revealed the variable reductions (~45.7%) in four mtDNA-encoded polypeptides in mutant cell lines. The impaired mitochondrial translation caused defects in respiratory capacity. Furthermore, defects in mitochondrial ATP synthesis and mitochondrial membrane potential (ΔΨm) were observed in mutant cell lines. These abnormalities resulted in the accumulation of oxidative damage and increasing of apoptosis in the mutant cell lines, as compared with controls. All those alterations may cause the primary degeneration of RGCs and subsequent visual loss. These data provided the direct evidence for c.1198C > G mutation leading to ADOA. Our findings may provide new insights into the understanding of pathophysiology of ADOA.

## Introduction

Autosomal dominant optic atrophy (ADOA, OMIM 165500) is the most common inherited optic neuropathies, affecting one individual per 12,000 to 35,000^1, 2^. This disorder is characterized by an insidious loss of visual acuity in early childhood with color vision deficits, central scotoma (blind spot) and temporal or diffuse pallor of the optic disc^3^. The typical features in this disorder involve the primary degeneration of RGCs, and accompanied by ascending optic atrophy^3–5^. The clear autosomal dominant pattern of inheritance and congenital nature of ADOA can be distinguished it from Leber hereditary optic atrophy (LHON), which exhibited the maternal inheritance and late-onset of optic atrophy and was caused by mutations in mitochondrial DNA (mtDNA)^5–8^. Mutations among five genes *OPA1*, *OPA3*, *OPA4*, *OPA5* and *OPA8* have been identified to be associated with ADOA^9–13^. Of these, the *OPA1* gene, encoding a mitochondrial dynamin-like GTPase protein, is the most prevalently causative gene for ADOA^3, 9, 10, 14^. About 350 variants of *OPA1* gene, consisting of 30 exons, were identified from various ethnic origins (http://mitodyn.org/home.php)^14^. The majority of *OPA1* mutations are located at the GTPase domain (Exons 8–15) and dynamin central regions (Exons 16–23)^14^. The primary defects of *OPA1* were the altered mitochondrial fusion and morphology^15–17^. These mutations also affected the mitochondrial genome maintenance^18, 19^. As a result, mutations in *OPA1* gene caused the deficient oxidative phosphorylation (OXPHOS) and sensitivity to apoptosis^20–24^.

Despite progress made in the identification of the genetic defects associated with ADOA in European and North American populations, less has been known about the pathogenesis of ADOA in the Asians, especially in the Chinese population. With aim to identify the molecular basis of ADOA in Chinese population, we recruited a large cohort of Chinese subjects with ADOA and LHON^6–8^. In our previous study, we reported a large four-generation Han Chinese family with ADOA^25^. Twelve of 44 family members were affected in this pedigree, and the ratio of affected male and female relatives was 1:1. The affected family members exhibited the early-onset and progressive visual impairment, ranging from mild to profound loss of visual acuity. The average age-at-onset was 14 years. The sequencing analysis of entire *OPA1* gene identified a novel c.1198C > G (p.P400A) heterozygous mutation at the exon 12 in all affected members of this family. The p.P400A mutation located at a highly conserved residue of GTPase domain of this protein. To further investigate the pathogenic mechanism of the c.1198C > G (p.P400A) mutation, lymphoblastoid cell lines were generated from 3 affected members carrying the c.1198C > G mutation and 3 control subjects lacking any of *OPA1* mutation. These cell lines were assessed for the effects of the *OPA1* mutation on mitochondrial morphology, mtDNA copy number, oxygen consumption ratio (OCR), ATP production, mitochondrial membrane potential, generation of reactive oxygen species (ROS) and sensitivity to apoptosis.

## Results

### The construction of lymphoblastoid cell lines

Immortalized lymphoblastoid cell lines used for biochemical assays were derived from three affected members carrying the c.1198C > G mutation and one vision normal member lacking the mutation of the family, and two genetically unrelated control individuals with the same mitochondrial background (Supplementary Figure 1a). The ages of four members (II-9, III-11, III-12 and IV-3) of this family were 47, 30, 27 and 6 years, respectively; while the ages of two Chinese control subjects (WZ11 and WZ12) were 42 and 8 years, respectively. The mtDNA haplogroups of these affected and control subjects belonged to Asian haplogroup M9. The Sanger sequence analysis confirmed the presence of the c.1198C > G heterozygous mutation in these cell lines derived from three relatives of this family but absence of the mutation in control subjects (Supplementary Figure 1b).

### Alteration in mitochondrial morphology

**Figure 1:**
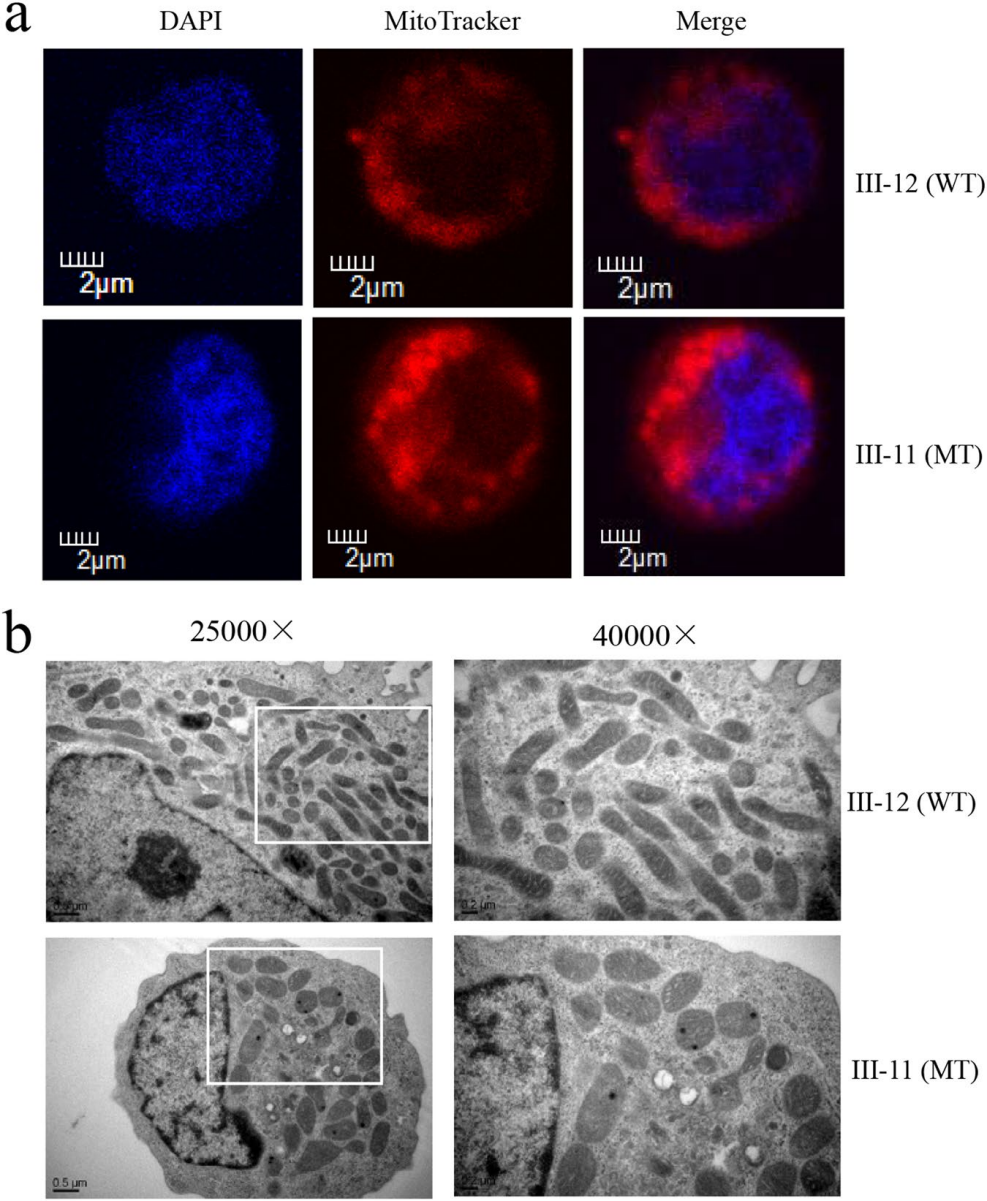
The *OPA1* mutation altered the fragmentation of the mitochondrial network. (**a**) Mitochondrial morphology was visualized by immunofluorescent labeling with MitoTracker (red) and was analyzed by confocal microscopy using lymphoblastoid cell lines. DAPI-stained nuclei were identified by their blue fluorescence. Internal control cell (III-12) lacking the *OPA1* mutation exhibited a balanced mitochondrial network of the filamentous and fragmented states. Cells carrying the *OPA1* mutation (III-11) displayed a distinct punctate pattern of mitochodnria. (**b**) Mitochondrial networks from patient and control cells were examined by transmission electron microscopy. Ultrathin sections were stained with uranyl acetate and alkaline lead citrate. 25,000X and 40,000X magnifications were used. Control lymphoblastoid cell lines (III-12) have tubular distribution of mitochondria. Patient-derived lymphoblastoid cell lines (III-11) have spotty distribution of mitochondria.

To assess the effects of the *OPA1* mutation on mitochondrial morphology, lymphoblastoid cell lines derived from one affected member (III-11) and one vision normal subject (III-12) were stained with mitochondrial-specific dye MitoTracker Red^TM^ and with DNA specific dye DAPI and analyzed with a confocal microscope system. As shown in Fig. 1a, the mitochondria in *OPA1* mutant cells (III-11) displayed a distinct punctate pattern, as compared with a balanced mitochondrial network in control cells (III-12). The mitochondria morphology from mutant and control lymphoblastoid cell lines was further examined by using transmission electron microscopy. As shown in Fig. 1b, the mutant cell lines showed the abnormal mitochondrial morphology including mitochondrial fragmentation, swollen and cristae structure, as compared with control cell lines. In particular, mutant cells exhibited a spotty distribution of mitochondria (5.9% with tubular and 94.1% with spotty) (spotty was defined as length of mitochondria < 0.60 μm, tubular as length > 0.60 μm), while the wild type cells displayed the tubular distribution of mitochondria (59.4% with tubular and 40.6% with spotty). In addition, the mitochondrial wide in the mutant cells were much larger than that of control cells (mutants 0.20–0.53 μm versus controls 0.13–0.33 μm). Furthermore, mitochondrial numbers in mutant cells ranged from 43.5% to 68.5%, with an average of 57%, related to average values of control cells. These results indicated that the c.1198C > G mutation resulted in the abnormality of mitochondrial morphology.

### Decrease in mtDNA copy number

**Figure 2:**
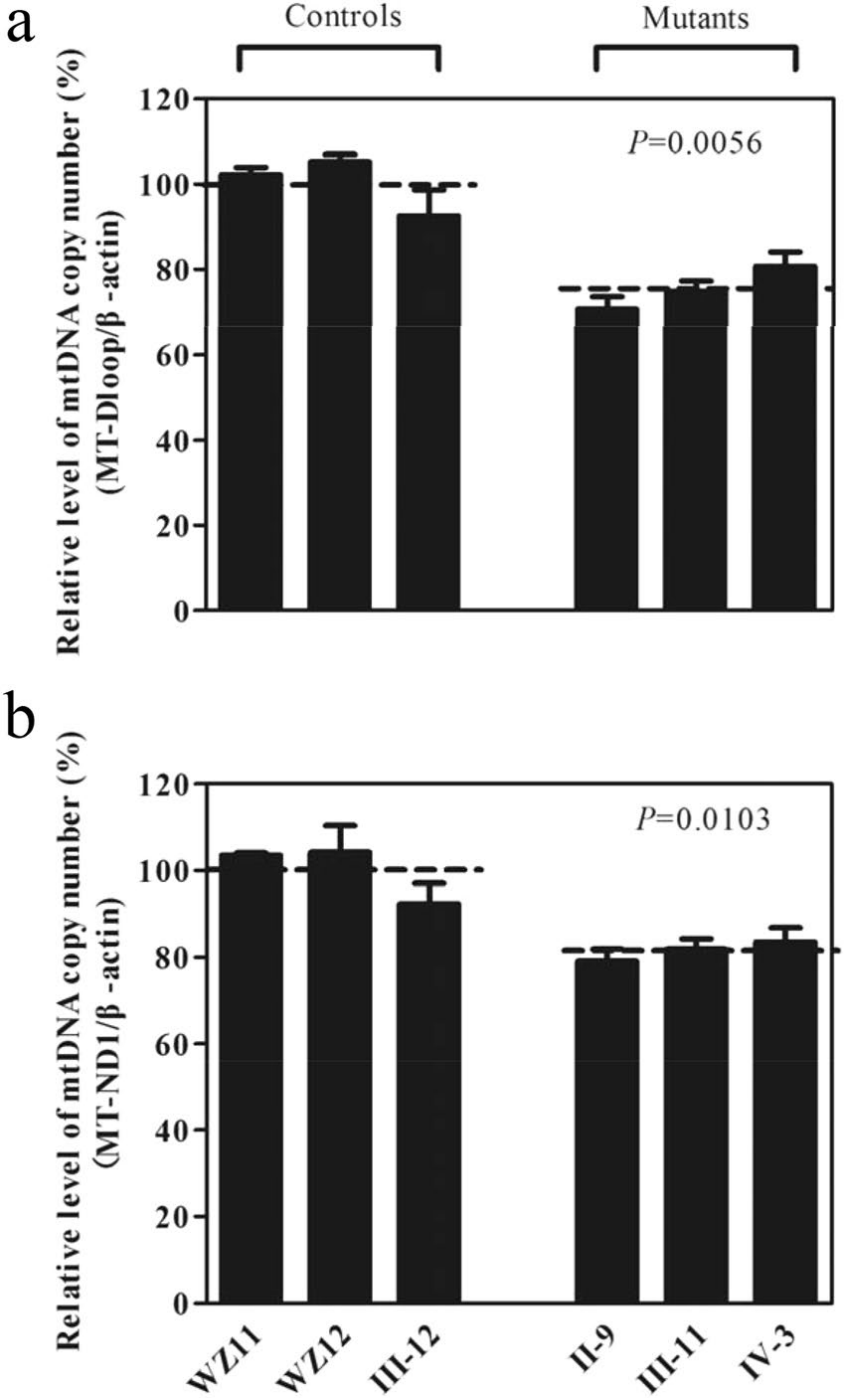
Measurements of mtDNA copy numbers in lymphoblastoid cell lines. The mtDNA copy numbers were determined by comparing the ratio of mtDNA to nDNA (β-actin) by real-time quantitative PCR. (**a**) The ratio of MT-D-loop region to β-actin. (**b**) The ratio of MT-ND1 to β-actin. The calculations were based on three independent determinations. The error bars indicate two standard errors of the mean (SEM). *P* indicates the significance, according to the Student t-test, of the differences between the mean of mutant and that of control cell lines.

It was anticipated that the alteration in maintenance of mitochondrial genome caused by mutation in *OPA* *1* affected the copy number of mtDNA^18^. Here, we examined the mtDNA copy number by quantitative PCR using total genomic DNA as template. For this purpose, we made standard curve of three pairs of primers (MT-D-loop, MT-ND1 and β-actin), as shown in the Supplemental Figure 2. In particular, mtDNA contents were measured using fragments spanning the MT-D-loop region and MT-ND1 as mtDNA targets and nuclear coding β-actin as the normalization. As shown in Fig. 2, the contents of mtDNA copies in mutant cell lines, using fragments spanning the D-loop region, ranged from 70.6% to 80.7%, with an average of 75.4% (*P* = 0.0056) and using fragments spanning MT-ND1 gene, from 79.1% to 83.5%, with an average of 81.5% (*P* = 0.0103), respectively, related to average values of control cell lines.

### Reduction in the level of mitochondrial proteins

**Figure 3:**
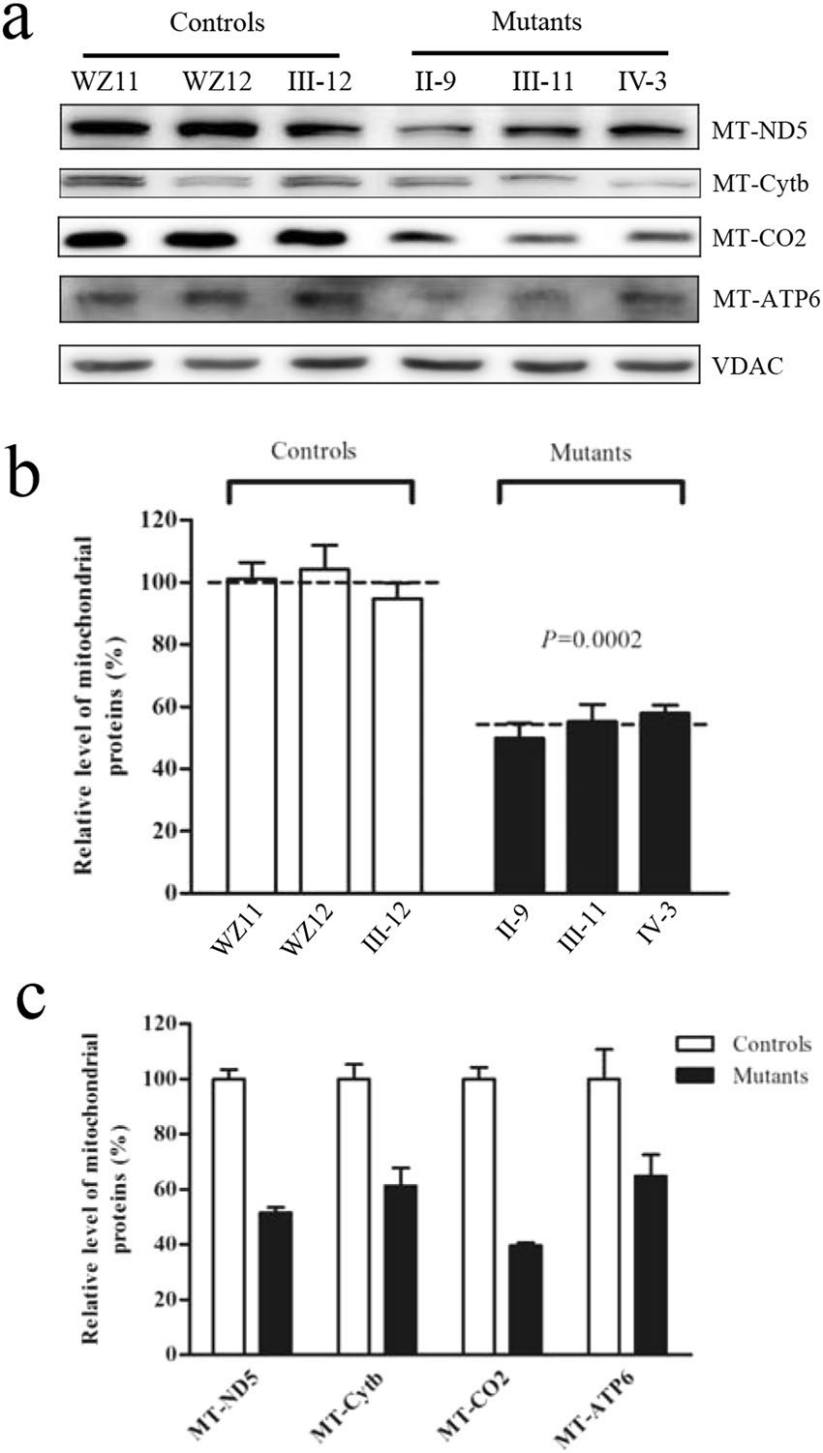
Western blot analysis of mitochondrial proteins. (**a**) Twenty microgram of total cellular proteins from various cell lines were electrophoresed through a denaturing polyacrylamide gel, electroblotted and hybridized with four respiratory complex subunits in mutants and controls with VDAC as a loading control. p.MT-ND5, subunits 5 of the reduced nicotinamide–adenine dinucleotide dehydrogenase; p.MT-CO2 indicate subunits 2 of cytochrome c oxidase; p.MT-Cytb, apocytochrome b; and p.MT-ATP6, subunit 6 of the H + -ATPase. (**b**) Quantification of total mitochondrial protein levels. The levels of mitochondrial proteins in 3 mutant cell lines and 3 control cell lines were determined as described elsewhere^27^. The values for the mutant cell lines are expressed as percentages of the average values for the control cell lines. The calculations were based on three independent determinations. Graph details and symbols are explained in the legend to Fig. 2. (**c**) Quantification of 4 respiratory complex subunits. The levels of p.MT-ND5, p.MT-Cytb, p.MT-CO2 and p.MT-ATP6 in 3 mutant cell lines and 3 control cell lines were determined as described elsewhere^27^.

To investigate whether the impairment of mitochondrial translation occurred in mutant cell lines, a Western blot analysis was carried out to examine the levels of four mtDNA-encoded respiratory complex subunits in mutant and control cell lines with a nuclear encoding mitochondrial protein VDAC as a loading control. As shown in Fig. 3a, the levels of p.MT-ND5 subunits 5 of NADH dehydrogenase; p.MT-Cytb, apocytochrome b; p.MT-CO2, subunits 2 of cytochrome c oxidase and p.MT-ATP6, subunit 6 of the H + -ATPase were decreased in three mutant cell lines, as compared with those of three control cell lines. As shown in Fig. 3b, the overall levels of four mitochondrial translation products in the mutant cell lines was decreased from 35.2% to 60.4%, with an average of 45.7% (*P* = 0.0002) of the man value ﻿measured in three control cell lines. Notably, the average levels of p.MT-ND5, p.MT-Cytb, p.MT-CO2 and p.MT-ATP6 in the mutant cell lines were 48.4%, 38.7%, 60.4% and 35.2% of the average values relative to the mean value measured in the control cell lines, respectively (Fig. 3c).

### Respiration deficiency

**Figure 4:**
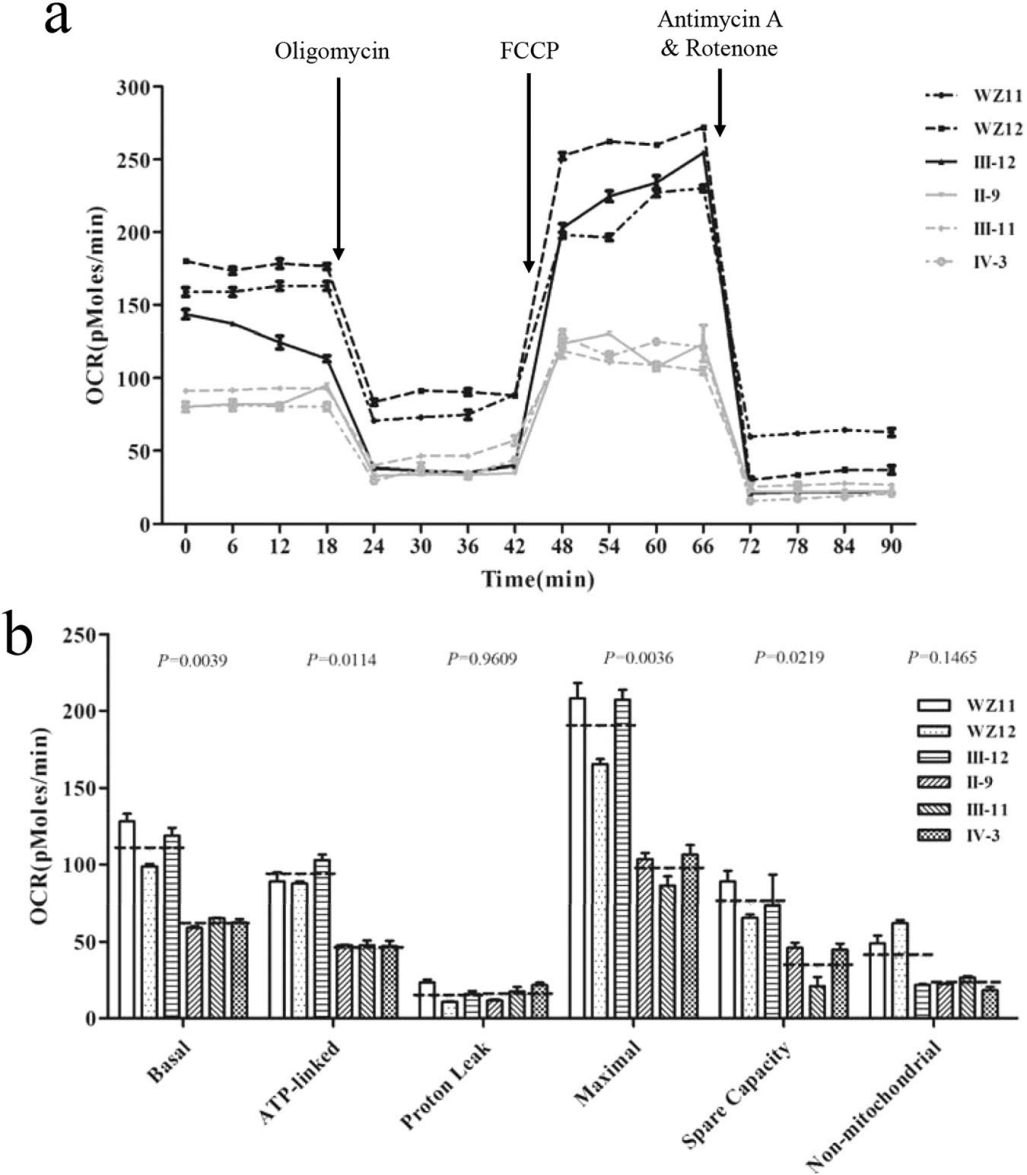
*OPA1* mutation impaired mitochondrial bioenergetics. (**a**) An analysis of O_2_ consumption in the various cell lines using different inhibitors. The rates of O_2_ (OCR) were first measured on 2 × 10^4^ cells of each cell line under basal condition and then sequentially added to oligomycin (1 μM), carbonyl cyanide p-(trifluoromethoxy) phenylhydrazone (FCCP) (0.5 μM), rotenone (1 μM) and antimycin A (1 μM) at indicated times to determine different parameters of mitochondrial functions. (**b**) Graphs presented the basal OCR, ATP-linked OCR, proton leak OCR, maximal OCR, reserve capacity OCR and non-mitochondrial OCR in mutant and control cell lines. Non-mitochondrial OCR was determined as the OCR after rotenone/antimycin A treatment. Basal OCR was determined as OCR before oligomycin minus OCR after rotenone/antimycin A. ATP-linked OCR was determined as OCR before oligomycin minus OCR after oligomycin. Proton leak was determined as basal OCR minus ATP-linked OCR. Maximal was determined as the OCR after FCCP minus non-mitochondrial OCR. Reserve capacity was defined as the difference between maximal OCR after FCCP minus basal OCR. The average of four determinations for each cell line is shown, the horizontal dashed lines represent the average value for each group. Graph details and symbols are explained in the legend to Fig. 2.

To evaluate if the c.1198C > G heterozygous mutation alters cellular bioenergetics, we measured the OCR of three mutant and three control cell lines using a Seahorse Bioscience XF-96 Extracellular Flux Analyzer. As shown in Fig. 4, the average basal OCR in three mutant cell lines was 53.9% (*P* = 0.0039) relative to the mean value measured in three control cell lines. To investigate which of the enzyme complexes of the respiratory chain was affected in the mutants, OCR were measured after the sequential addition of oligomycin (inhibit the ATP synthase), FCCP (to uncouple the mitochondrial inner membrane and allow for maximum electron flux through the electron transport chain), rotenone (to inhibit complex I) and antimycin A (to inhibit complex III)^26–28^. The difference between the basal OCR and the drug-insensitive OCR yields the amount of ATP-linked OCR, proton leak OCR, maximal OCR, reserve capacity and non-mitochondrial OCR. As shown in Fig. 4, the ATP-linked OCR, proton leak OCR, maximal OCR, reserve capacity and non-mitochondrial OCR in mutant cell lines were ~49.0%, 1.5%, 49.0%, 51.0% and 49.1%, relative to the mean value measured in the three control cell lines (*P* = 0.0114, 0.9606, 0.0036, 0.0219 and 0.1465), respectively.

### Impaired mitochondrial ATP synthesis

**Figure 5:**
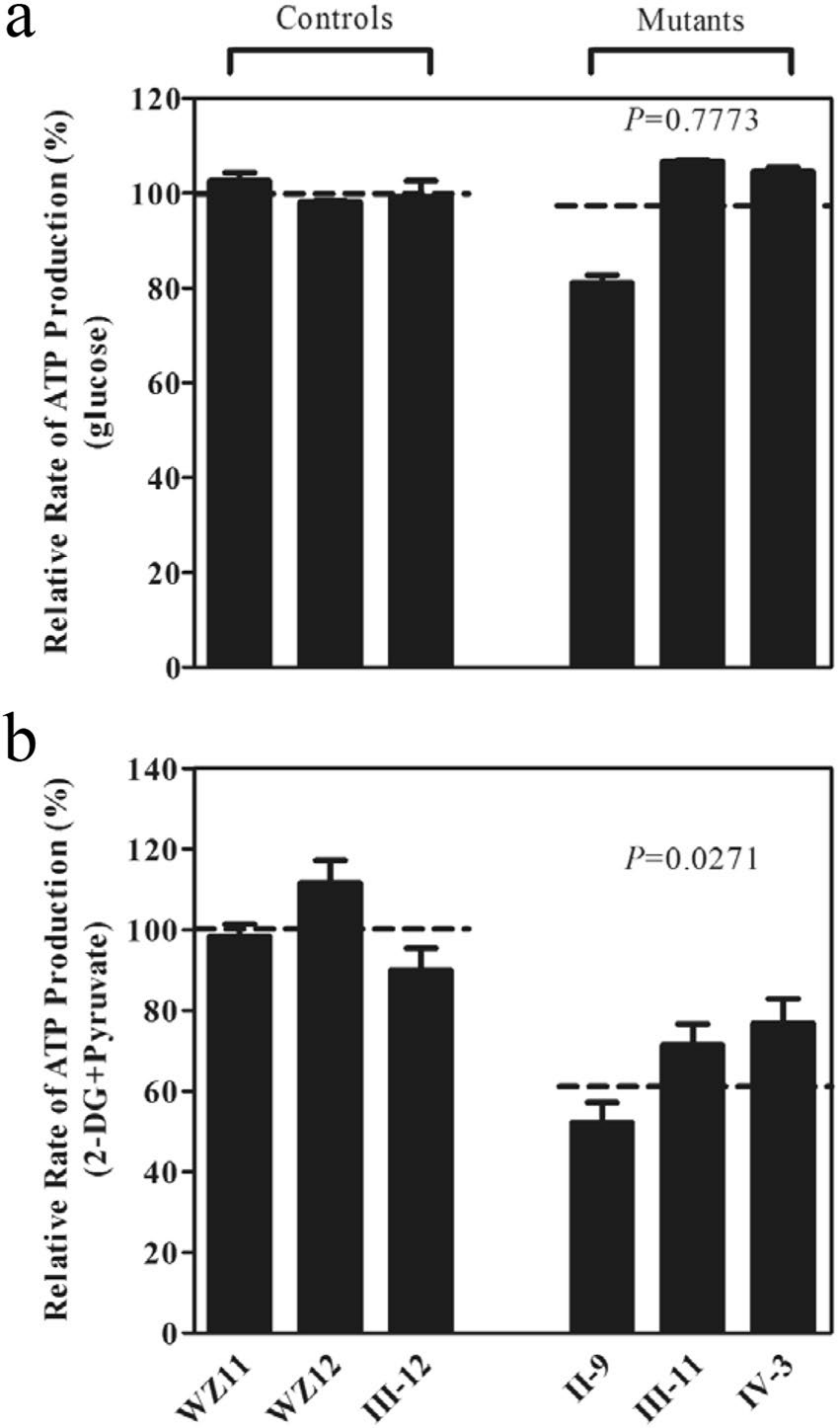
Measurement of cellular and mitochondrial ATP levels using bioluminescence assay. Cells were incubated with 10 mM glucose or 5 mM 2-deoxy-D-glucose plus 5 mM pyruvate to determine ATP generation under cellular and mitochondrial ATP levels. Average rates of ATP level per cell line are shown: (**a**) ATP level in total cells and (**b**) ATP level in mitochondria. Six to seven determinations were made for each cell line. Graph details and symbols are explained in the legend to Fig. 2.

The capacity of oxidative phosphorylation in mutant and control cells was examined by measuring the levels of cellular ATP using a luciferin/luciferase assay. Populations of cells were incubated in the media in the presence of glucose, glucose with oligomycin, and 2-deoxy-D-glucose (2-DG) with pyruvate^27–29^. The levels of ATP production in mutant cell lines in the presence of glucose (total cellular levels of ATP) or 2-DG with pyruvate to inhibit the glycolysis (oxidative phosphorylation) were comparable with those measured in the control cell lines. By contrast, total cellular levels of ATP have no difference between mutant and control cell lines (Fig. 5a), while the levels of ATP production from oxidative phosphorylation in the mutant cell lines, ranged from 52.1% to 76.8%, with an average of 66.8% relative to the mean value measured in three control cell lines (*P* = 0.0271) (Fig. 5b).

### Decrease in mitochondrial membrane potential

**Figure 6:**
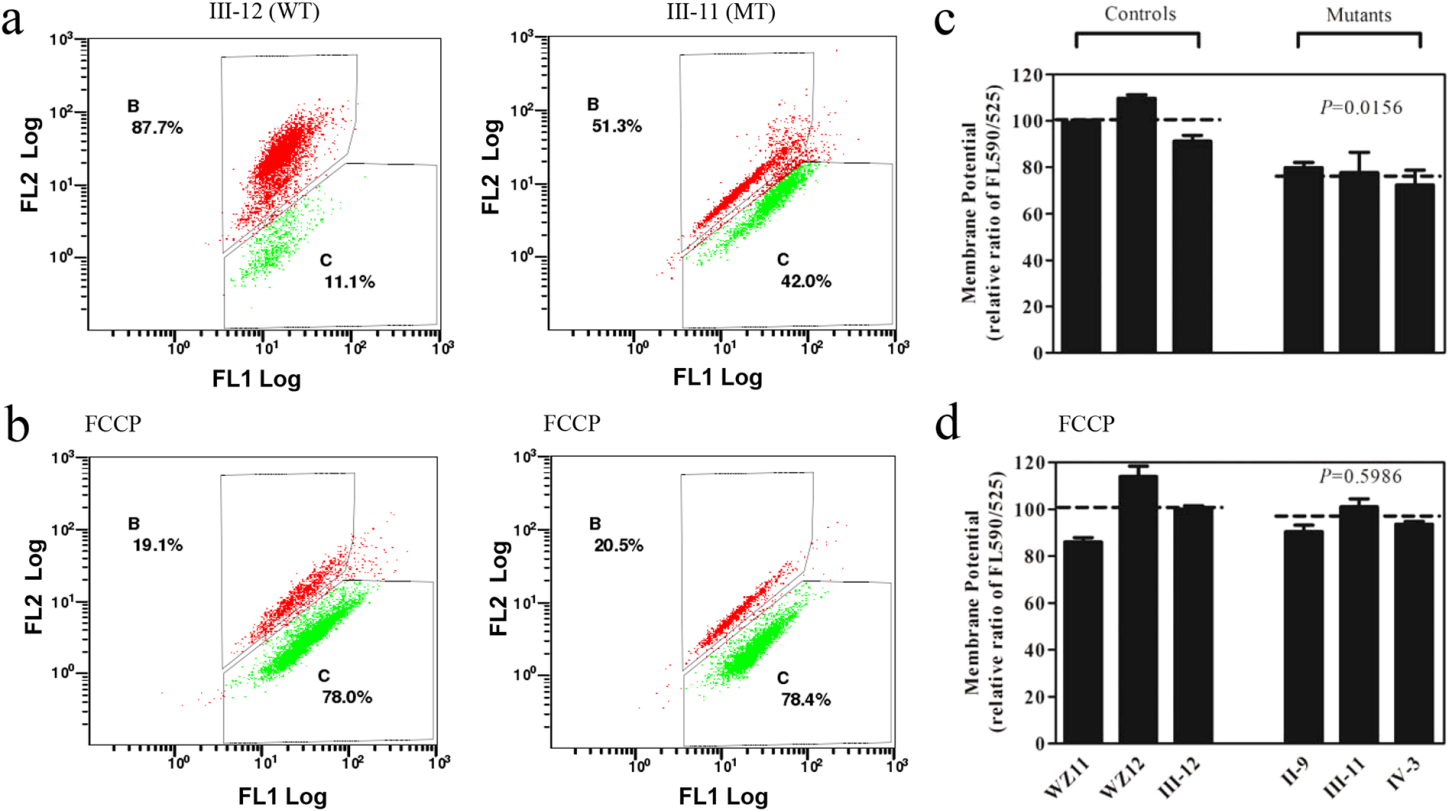
Mitochondrial membrane potential analysis. The mitochondrial membrane potential (ΔΨm) was measured in three mutant and three control cell lines via the JC-10 assay. The ratio of the Ex/Em = 488/590 nm and 488/525 nm fluorescence intensities (FL590/FL525) was recorded to determine the ΔΨm level of each sample. The relative ratios of the FL590/FL525 geometric means between the mutant and control cell lines were calculated to reflect the level of ΔΨm. Flow cytometry images of III-12 and III-11 with (**a**,**b**) and without (**d**,**e**) FCCP. Relative ratio of JC-10 fluorescence intensities at Ex/Em = 490/525 and 490/590 nm in absence (**c**) and presence (**f**) of 10 mM of FCCP. The average of three to five determinations for each cell line is shown. Graph details and symbols are explained in the legend to Fig. 2.

The mitochondrial membrane potential (ΔΨm) alterations were measured using a fluorescence probe JC-10 assay system. The ratio of fluorescence intensities Ex/Em = 490/590 and 490/525 nm (FL590/FL525) were recorded to delineate the ΔΨm level of each sample. The relative ratios of FL590/FL525 geometric mean between mutant and control cell lines were calculated to represent the level of ΔΨm. As shown in Fig. 6, levels of the ΔΨm in the mutant cell lines carrying c.1198C > G mutation were decreased, ranging from 72.2% to 79.9%, with an average 76.5% (*P* = 0.0156) of the mean value measured in three control cell lines. By contrast, the levels of ΔΨm in mutant cell lines in the presence of FCCP were comparable with those measured in the control cell lines (*P* = 0.5986).

### The increase of ROS production

**Figure 7:**
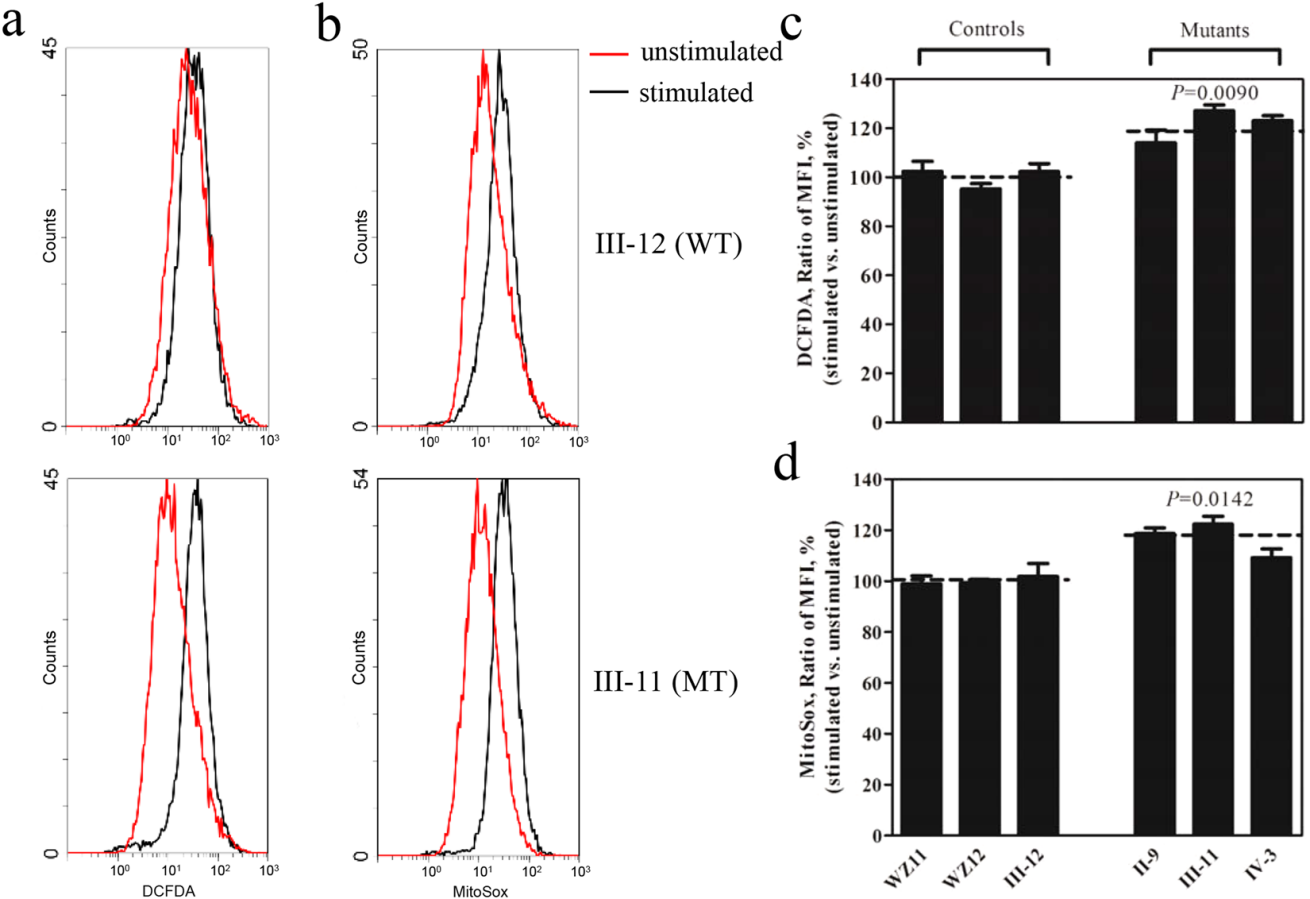
Ratio of geometric mean intensity between levels of the ROS generation in the vital cells with or without H_2_O_2_ stimulation. The rates of production in ROS from three mutant cell lines and three control cell lines were analyzed by FC500MCL flow cytometer system (Beckman) using (**a**) DCFDA (20 mM) and (**b**) MitoSox (5 mM), either without (Red) or with (Black) H_2_O_2_ stimulation. The relative ratio of intensity (stimulated versus unstimulated with H_2_O_2_) was calculated. (**c**) Total ROS generation in the vital cells and (**d**) mitochondrial ROS generation. The average of three independent determinations for each cell lines is shown. Graph details and symbols are explained in the legend to Fig. 2.

The levels of the ROS generation in the vital cells derived from three affected relatives carrying the c.1198C > G mutation and three control individuals lacking this mutation were measured with flow cytometry under normal and H_2_O_2_ stimulation^29, 30^. Geometric mean intensity was recorded to measure the rate of ROS in each sample. The ratio of geometric mean intensity between unstimulated and stimulated cell lines with H_2_O_2_ was calculated to delineate the reaction upon increasing level of ROS under oxidative stress. As shown in Fig. 7a and c, the levels of ROS generation in the lymphoblastoid cell lines derived from three affected individuals carrying the c.1198C>G mutation were reduced, ranging from 114.1% to 127.1%, with an average of 121.4% (*P* = 0.0090) of the mean value measured in three control cell lines. Furthermore, the levels of mitochondrial ROS (mito-ROS) among lymphoblastoid cell lines were determined using a MitoSOX assay via flow cytometry^31, 32^. As show in Fig. 7b and d, the levels of mitochondrial ROS generation in the mutant cell lines ranged from 109.2% to 122.3%, with an average of 116.7%(*P* = 0.0142) of the man value measured in three control cell lines.

### Defects in cell apoptosis

**Figure 8:**
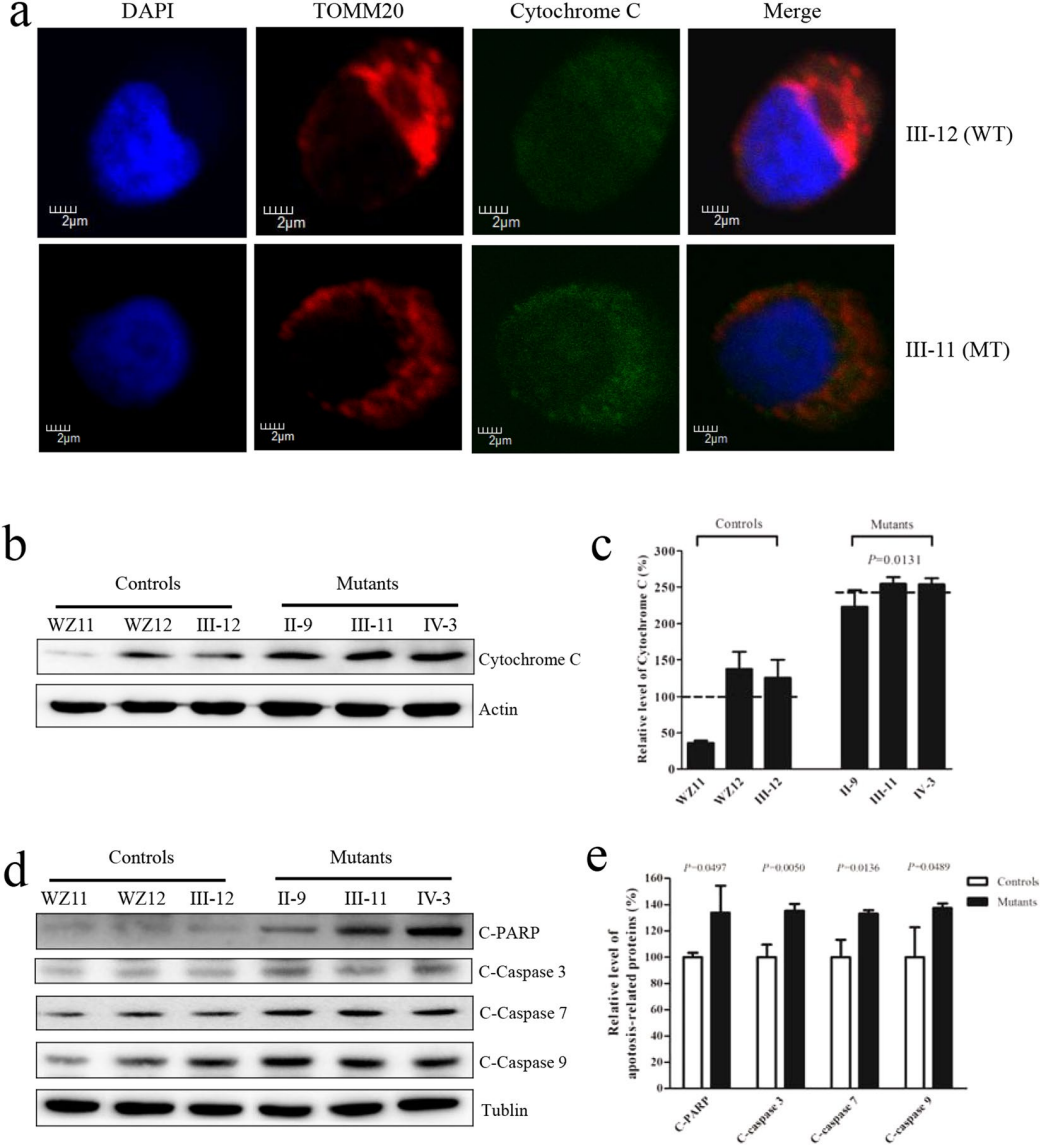
*OPA1* mutation triggers apoptosis. (**a**) The distributions of cytochrome c from lymphoblastoid cell lines [patient III-11 and internal control III-12] were visualized by immunofluorescent labeling with an antimitochondrial TOM20 antibody conjugated to Alex Fluor 594 (red) and cytochrome c antibody conjugated to Alex Fluor 488 (green) was analyzed by confocal microscopy. DAPI-stained nuclei were identified by their blue fluorescence. (**b**) 20 μg of total proteins from various cell lines were electrophoresed through a denaturing polyacrylamide gel, electroblotted and hybridized with cytochrome c with β-actin as a loading control. (**c**) Quantification of cytochrome c. The levels of cytochrome c in mutant and control cell lines were determined as described elsewhere^28^. The calculations were based on three independent determinations in each cell line. (**d**) 20 μg of total proteins from various cell lines were electrophoresed through a denaturing polyacrylamide gel, electroblotted and hybridized with PARP, Caspases 3, 7 and 9 with Tublin as a loading control. (**e**) Quantification of four caspases. The levels of PARP, Caspases 3, 7 and 9 in mutant and control cell lines were determined as described elsewhere^28^. The calculations were based on three independent determinations in each cell line. Graph details and symbols are explained in the legend to Fig. 2.

To evaluate if the c.1198C > G mutation affects the apoptotic process, we examined the apoptotic state of mutant and control lymphoblastoid cell lines by using immunocytostaining with the antibodies and analyzed with a confocal microscope system and Western blotting analysis. Figure 8a shows the immunofluorescence pattern of lymphoblastoid cell lines double labeled with rabbit monoclonal antibody specific for the Cytochrome C and mouse monoclonal antibody to TOM20, a nuclear encoding protein in the mitochondrial inner membrane. As shown in Fig. 8b and c, the levels of cytochrome c in the mutant cell lines increased 143.7%, as compared with the average values in control cell lines. To further analyze if the c.1198C > G mutation affects apoptosis, we measured the levels of apoptosis-related proteins: PARP, as well as caspases 3, 7 and 9 in mutant and control cell lines by Western blot analysis. As showed in Fig. 8d and e, the average levels of PARP protein, caspase 3, caspase 7 and caspase 9 in the mutant cell lines were 133.7%, 135.3%, 133.1% and 137.3% of the average values measured in the control cell lines, respectively (*P* = 0.0497, 0.0050, 0.0136 and 0.0489).

## Discussion

The *OPA1* gene encoding a mitochondrial dynamin-like GTPase protein is the most important causative gene for ADOA. Indeed, OPA1 is a critical factor connecting the mitochondrial morphology and energetics. In this investigation, we characterized the novel c.1198C > G (p. P400A) mutation in *OPA1* gene using lymphoblastoid cell lines generated from members of a large Chinese family with ADOA. The p. P400A mutation was located at a highly conserved residue of GTPase domain where the majority of pathogenic mutations in *OPA1* gene cluster^14^. The G401 residue is one of the key residues in the G3 (DxxG) motif and may form a hydrogen bond with the γ-phosphate of GTP^15^. Furthermore, the p.P400A mutation may also cause misalignment of GTP in the GTP-binding pocket of the GTPase domain of *OPA1*, resulting in mitochondrial dysfunction^25^. It was anticipated that the p.P400A mutation caused the mitochondrial fragmentation, instability of mtDNA, and OXPHOS defect. The primary defects in the mutations of *OPA1* gene appeared to alter the mitochondrial morphology and fusion^16, 33–36^. In the present study, mutant lymphoblastoid cell lines carrying the heterozygous c.1198C > G mutation revealed the altered mitochondrial dynamics and led to the fragmented network, compared with control cell lines. In particular, the mutant cells exhibited the perturbed mitochondrial morphology including mitochondrial fragmentation, swollen and cristae structure and spotted distribution of mitochondria, as in the case of cell lines carrying the other OPA1 mutations^16, 36^. In fact, the altered fusion activity and the increase in mitochondrial network fragmentation in mutant cells carrying the c.1198C > G mutation may be due to the dominant negative effect^17^.

The OPA1/MGM1 are required for the maintenance of mitochondrial genomes^18^. The mutations in *OPA1* gene led to the depletion of mtDNA in cells and skeletal muscular tissues derived from ADOA subjects^37, 38^. In the present study, 24.6% decreases in mtDNA content observed in mutant cells were due to the unstable maintenance of mtDNA caused by the *OPA1* mutation. The unstablity of mtDNA caused by the *OPA1* mutation contributed to the deficient mitochondrial translation. In the present study, a markedly decreased level of mitochondrial proteins (an average decrease of ~45.7%) was observed in mutant cells, as compared to the average levels in control cells. The reduced level of variable mitochondrial proteins detected in lymphoblastoid cell lines using a Western blot analysis was comparable with the reduced rate of mitochondrial protein synthesis observed in cell lines carrying mtDNA mutations^27, 31^. The impairment of mitochondrial translation then resulted in the respiration defects. In particular, alteration in mitochondrial protein synthesis was apparently responsible for the significant reduced rates in the basal OCR, ATP-linked OCR, maximal OCR and reserve capacity OCR in the control and mutant cell lines. This correlation is clearly consistent with the importance that *OPA1* mutation plays a critical role in producing their respiration defects, as in the cases of cells both carrying the homozygous *YARS2* c.572G > T and LHON-associated m.11778G > A mutations^28^. The respiratory deficiency then affects the efficiency of mitochondrial ATP synthesis. Our study showed that these lymphoblastoid cell lines carrying the *OPA1* mutation had 33.2% decrease in the mitochondrial ATP production. These results were consistent with other report, showing significant reductions in the ATP content in cell lines carrying the other *OPA1* mutations^18^. Furthermore, our previous investigations revealed the marked decreases in mitochondrial ATP production in lymphoblastoid cell lines derived from Chinese pedigrees with LHON^28, 29^. Alternatively, the reduction in mitochondrial ATP production in mutant cells was likely a consequence of the decrease in the proton electrochemical potential gradient of mutant mitochondria^39^. As a result, RGC cells carrying the *OPA1* mutation may be particularly sensitive to decreased ATP demand. Furthermore, the deficient activities of respiratory chain complexes often alter mitochondrial membrane potentials, which indicated the viability of cells^40^. In this investigation, 24% reduction in mitochondrial membrane potential in lymphoblastoid cell lines carrying the *OPA1* mutation was observed, which was lower than those in cell lines carrying the m.12201T > C mutation in the tRNA^His gene 27^. The defects in mitochondrial membrane potential may be due to strongly decreased efficiency of respiratory chain mediated proto extrusion for the matrix. The impairment of OXPHOS can lead to more electron leakage from electron transport chain, and in turn, elevate the production of ROS in mutant cells^41^. In the present study, the levels of ROS generation in the lymphoblastoid cell lines derived from three affected individuals carrying this mutation were 21.4% higher than control cell lines, and mitochondrial ROS generation increased 16.7% in the mutants, while 47% increase of ROS production in cells carrying both m.11778G > A and homozygous *YARS2* c.572G > T mutations was the consequence of the altered activities of complexes I and IV^28^. The overproduction of ROS can establish a vicious cycle of oxidative stress in the mitochondria, thereby damaging mitochondrial and cellular proteins, lipids and nuclear acids^42^.

Apoptosis is the final common pathway leading to RGCs loss in ADOA and cell death is likely being complex, being triggered by a combination of several interacting factors. The two best-understood activation mechanisms are the intrinsic pathway (also called the mitochondrial pathway) and the extrinsic pathway^43, 44^. At a molecular level, apoptosis is regulated by two protein families: the Bcl-2 family which is responsible for the initiation phase, and the caspase family of proteases are that are involved in the execution phase of apoptosis^45, 46^. Mitochondrial cytochrome c plays an important role in the propagation of many pro-apoptotic signals through acting as a co-factor for a caspase-activating complex in the cytoplasm, called the apoptosome. As a result, release of cytochrome c from the mitochondrial intermembrane space (IMS) represents an important checkpoint in apoptosis^42, 47^. Downregulation of *OPA1* leads to aberrant cristae remodeling and the release of cytochrome c which is normally sequestered within the tight cristae junctions^23^. In the present investigation, immunocytostaining on cytosolic and mitochondrial fractions showed that the levels of cytochrome c in mutant cell lines was higher than in control cell lines. Furthermore, Western blot analysis demonstrated that levels of the cytochrome c mutants exhibited markedly higher than those in control cell lines. In addition, the levels of apoptosis-related proteins: PARP, as well as caspases 3, 7 and 9 in the mutant cell lines were significantly higher those in control cell lines. These data demonstrated that c.1198G > C mutation in the *OPA1* gene increased apoptosis, especially in the RGCs carrying c.1198C > G mutation. As a result, mitochondrial dysfunction caused by the c.1198G > C mutation in the *OPA1* gene produced a phenotype of visual loss in this Chinese family.

In summary, our findings suggest the pathogenic mechanism leading to an impaired oxidative phosphorylation in lymphoblastoid cell lines cell lines carrying the c.1198C > G mutation in the *OPA1* gene. The c.1198C > G mutation alters the mitochondrial morphology and mitochondrial genome maintenance. These defects impaired mitochondrial translation and respiration. As a result, this respiratory deficiency reduced mitochondrial ATP production, increased the production of oxidative reactive species, elevated apoptosis of RGCs and subsequently produced a phenotype of visual loss. Thus, our findings may provide new insights into the understanding of pathophysiology of ADOA.

## Materials and Methods

### Cell cultures and culture conditions

This study was in compliance with the Declaration of Helsinki. Informed consent, blood samples, and clinical evaluations were obtained from all participating family members, under protocols approved by the Ethic Committees of Zhejiang University and the Wenzhou Medical University. Lymphoblastoid cell lines derived from four members of this Chinese ADOA family 3 vision-impaired members (II-9, III-11, IV-3) carrying the *OPA1* c.1198C > G mutation, vision normal member (III-12) lacking the c.1198C > G mutation^25^, and from two genetically unrelated control individuals (WZ11 and WZ12) lacking any *OPA1* mutation but belonging to the same mtDNA haplogroup were immortalized by transformation with the Epstein-Barr virus, as described previously^48^. Cell lines were grown in RPMI1640 medium (Invitrogen), supplemented with 10% fetal bovine serum.

### Mitochondrial network analysis

Cells were cultured on collagen-coated cover glass slips (Thermo Fisher) for 24 hours in the presence of serum. To visualize the mitochondrial network, MitoTracker Red^TM^ (Invitrogen) and 4′, 6-diamidino-2-phenylindole (DAPI; Invitrogen) were employed. The cells grown on cover slips were incubated in growth medium supplemented with 80 nM MitoTracker Red^TM^ for 30 min, stained with DAPI for 15 minutes, and then washed in warm fresh medium, mounted. Cells were examined using a confocal fluorescence microscope (Olympus Flu view FV1000, Japan) with two lasers (Ex/Em = 579/599 nm and 358/461 nm). Mitochondrial morphology was determined in 100 individual cells.

### Immunocytochemistry Analysis

Triple fluorescent staining was performed to detect translocase of outer mitochondrial membrane 20 (TOMM20; Proteintech Group, USA), cytochrome C (Abcam) and nuclei, using 4′, 6-diamidino-2-phenylindole (DAPI; Invitrogen, USA). Cells were cultured on collagen-coated cover glass slips (Thermo Fisher), fixed in 4% formaldehyde for 15 minutes, permeabilized with 0.2% Triton X-100, blocked with 5% Fetal Bovine Serum (FBS) for 1 hour, and immunostained with an anti-TOMM20 antibody and anti-cytochrome C overnight at 4 °C. The cells were then incubated with Alex Fluor 594 goat anti-rabbit IgG (H + L) and Alex Fluor 488 goat anti-mouse IgG (H + L) (Thermo Fisher), stained with DAPI for 15 minutes, and mounted with Fluoromount (Sigma-Aldrich). Cells were examined using a confocal fluorescence microscope (Olympus Fluoview FV1000, Japan) with three lasers (Ex/Em = 550/570 nm, 492/520 nm and 358/461 nm).

### Transmission electron microscopy

Specimens were fixed with 2.5% glutaraldehyde in phosphate buffer (0.1 M, pH: 7.0) for more than 4 hours, followed by 1% osmium tetroxide in phosphate buffer (0.1 M, pH: 7.0) for 1–2 hours. Specimens were then dehydrated by a graded series of ethanol, infiltration, and embedded in epoxy resin. Ultrathin sections were stained with uranyl acetate and alkaline lead citrate for 5–10 min respectively and viewed with Hitachi Model H-7650 TEM.

### Quantification of mtDNA copy number

Total genomic DNAs were extracted with the MiniBEST Universal Genomic DNA Extraction Kit (TaKaRa) from mutant and control cell lines. 50 ng of DNA was used in each QPCR reaction using Universal SYBR Green Master (Roche Applied Science, Mannheim, Germany) with two pairs of primers for mtDNA (300 nM each) in a 7900 HT Fast Real-time PCR System (Applied Biosystems) with one pair of primers for β-Actin for normalization. The following primers were used: MT-D-loop region: 5′-TCACCCTATTAACCACTCA-3′ (sense) and 5′-AGACAGATACTGCGACATA-3′ (anti-sense); MT-ND1: 5′-CACCCAAGAACAGGGTTTGT-3′ (sense) and 5′-TGGCCATGGGTATGTTGTTAA-3′ (anti-sense); β-Actin: 5′-TCACCCACACTGTGCCCATCTACGA-3′ (sense) and 5′-CAGCGGAACCGCTCATTGCCAATGG-3′ (antisense). mtDNA contents were calculated using the ΔΔCt method whereby all MT-D-loop and MT-ND1 (mtDNA target) Ct values were first normalized to β-Actin. Data from multiple experiments were analyzed using the procedure as described elsewhere^49^.

### Western blotting analysis

Western blotting analysis was performed as detailed previously^27, 28^. Twenty micrograms of cellular proteins obtained from lysed cells were denatured and loaded on sodium dodecyl sulfate (SDS) polyacrylamide gels. Afterward, the gels were electroblotted onto a polyvinylidene difluoride (PVDF) membrane for hybridization. The antibodies used for this investigation were from Abcam [Anti-ND5, Anti-CO2, Anti-ATP6, VDAC, TOM20, Anti-Cytochrome c], Santa Cruz [Anti-Cytb]; CST [Cleaved PARP, Cleaved Caspase-3, Cleaved Caspase-7, Cleaved Caspase-9]. Peroxidase Affini Pure goat anti-mouse IgG and goat anti-rabbit IgG (Jackson) were used as a secondary antibody and protein signals were detected using the ECL system (CWBIO). Quantification of density in each band was performed as detailed previously^27, 28^.

### Mitochondrial respiration extracellular flux analysis

The rates of oxygen consumption in lymphoblastoid cell lines were measured with a Seahorse Bioscience XF-96 Extracellular Flux Analyzer (Seahorse Bioscience), as detailed previously^26, 27^. XF96 creates a transient, 7 μl-chamber in specialized microplates that allows for the determination of oxygen and proton concentrations in real time. To allow comparison between different experiments, data are expressed as the rate of oxygen consumption in pmol/min or the rate of extracellular acidification in mpH/min, normalized to cell protein in individual wells determined by the Bradford protein assay (Bio-Rad). A density of 1 × 10^5^ cells per well in 96-well plate was coated with Cell-Tak^TM^ adhesive. The rates of O_2_ were determined under basal condition and the addition of oligomycin (1 μM), carbonyl cyanide p-(trifluoromethoxy) phenylhydrazone (FCCP) (0.5 μM), rotenone (1 μM) and antimycin A (1 μM), as detailed elsewhere^26, 27^.

### Measurement of intracellular ATP levels

The levels of cellular and mitochondrial ATP levels were measured using the ATP Bioluminescence Assay Kit HS II (Roche Applied Science, Mannheim, Germany) according to the manufacturer’s instruction^27, 28^. In brief, samples of 2 × 10^6^ cells were incubated for 2 hours in the record solution (156 mM NaCl, 3 mM KCl, 2 mM MgSO_4_, 1.25 mM KH_2_PO_4_, 2 mM CaCl_2_, 20 mM HEPES, pH 7.35) with 10 mM glucose, 10 mM glucose plus 2.5 μg/mL oligomycin (glycolytic ATP generation), or 5 mM 2-deoxy-D-glucose plus 5 mM pyruvate (oxidative ATP production). Cells were lysed and then incubated with the luciferin/luciferase reagents. After 10 min incubation in room temperature, the luminescences were read on a microplate reader (Syneregy H1, Bio-Tek).

### Assessment of mitochondrial membrane potential

Mitochondrial membrane potential was assessed with JC-10 Assay Kit Flow Cytometry (Abcam) following general manufacturer’s recommendations with some modifications^27^. In brief, cells were plated onto 96-well cell culture plate overnight in growth medium. JC-10 dyeloading solution was added for 30 min at 37 °C, 5% CO_2_. Alternatively, plated cells were preincubated with 10 μM of the protonophore uncoupler carbonyl cyanide 3-chlorophenylhydrazone (CCCP) for 30 min at 37 °C, 5% CO_2_ prior to staining with JC-10 dye. The fluorescent intensities for both J-aggregates and monomeric forms of JC-10 were analyzed by BD-LSR II flow cytometer system (Beckton Dickson, Inc.) at Ex/Em = 490/525 and 490/590 nm. Ten thousand events were analyzed in each sample.

### ROS Measurements

ROS measurements were performed following the procedures detailed previously^30, 50^. Briefly, approximate 2 × 10^6^ cells of each cell line were harvested, resuspended in PBS supplemented with 100 μM of 2′,7′- dichlorodihydrofluorescein diacetate (DCFH-DA) and then incubated at 37 °C for 20 min. After washing with PBS twice, cells were resuspended in PBS in the presence of 2 mM freshly prepared H_2_O_2_ and 2% FBS and then incubated at room temperature for another 45 min. Cells were further washed with PBS and resuspended with 1 ml of PBS with 0.5% paraformaldehyde. Samples with or without H_2_O_2_ stimulation were analyzed by BD-LSR II flow cytometer system (Beckton Dickson, Inc.), with an excitation at 488 nm and emission at 529 nm. Ten thousand events were analyzed in each sample. The levels of ROS generation by mitochondria in living cells were analyzed using the mitochondrial superoxide indictor MitoSOX-Red (Invitrogen), as detailed elsewhere^31^.

### Statistics

All statistical analyses were performed using Prism software (GraphPad Prism). Significance between two sets of experiments was determined using a Student’s t-test whereas group sets were analyzed using ANOVA. Differences were considered statistically significant when *P* value < 0.05.

## Electronic supplementary material

Suppelmental inforamtion
