# Supplementary material for: A novel ADOA-associated OPA1 mutation alters the mitochondrial function, membrane potential, ROS production and apoptosis
Source: Sci Rep. 2017 Jul 18;7:5704. doi: 10.1038/s41598-017-05571-y (PMC5515948; doi:10.1038/s41598-017-05571-y)
Supplement: Supplementary file 1 — Suppelmental inforamtion [file 41598_2017_5571_MOESM1_ESM.pdf]

# A novel ADOA-associated *OPA1* mutation alters the mitochondrial function, membrane potential, ROS production and apoptosis

Juanjuan Zhang[1,2,3,+], Xiaoling Liu[3,+], Xiaoyang Liang[2], Yuanyuan Lu[4], Ling Zhu[1,2], Runing Fu[3,4], Yanchun Ji[1,2], Wenlu Fan[3], Jie Chen[3], Bing Lin[3], Yimin Yuan[3,4], Pingping Jiang[1,2,], Xiangtian Zhou[3,*] and Min-Xin Guan[1,2,5,*]

[1]Division of Medical Genetics and Genomics, Zhejiang Children's Hospital, Zhejiang University School of Medicine, Hangzhou, Zhejiang, 310058 China.

[2]Institute of Genetics, Zhejiang University School of Medicine, Hangzhou, Zhejiang, 310058 China.

[3]School of Ophthalmology and Optometry, Wenzhou Medical University, Wenzhou, Zhejiang, 325027 China.

[4]Attardi Institute of Mitochondrial Biomedicine, Wenzhou Medical University, Wenzhou, Zhejiang, 325035 China.

[5]Collaborative Innovation Center for Diagnosis and Treatment of Infectious Diseases, Zhejiang University, Hangzhou, Zhejiang 310058, China

***To whom correspondences should be:** Min-Xin Guan, Ph.D, Institute of Genetics, Zhejiang University School of Medicine, Hangzhou, Zhejiang, China; Telephone: (571) 88206916; Fax: (571) 88982377; E-mail: gminxin88@zju.edu.cn.

Xiangtian Zhou, MD, School of Ophthalmology and Optometry, Wenzhou Medical University, Wenzhou, Zhejiang, China, E-mail: zxt-dr@wz.zj.cn.

[+]J. Zhang and X. Liu contributed equally to this work.

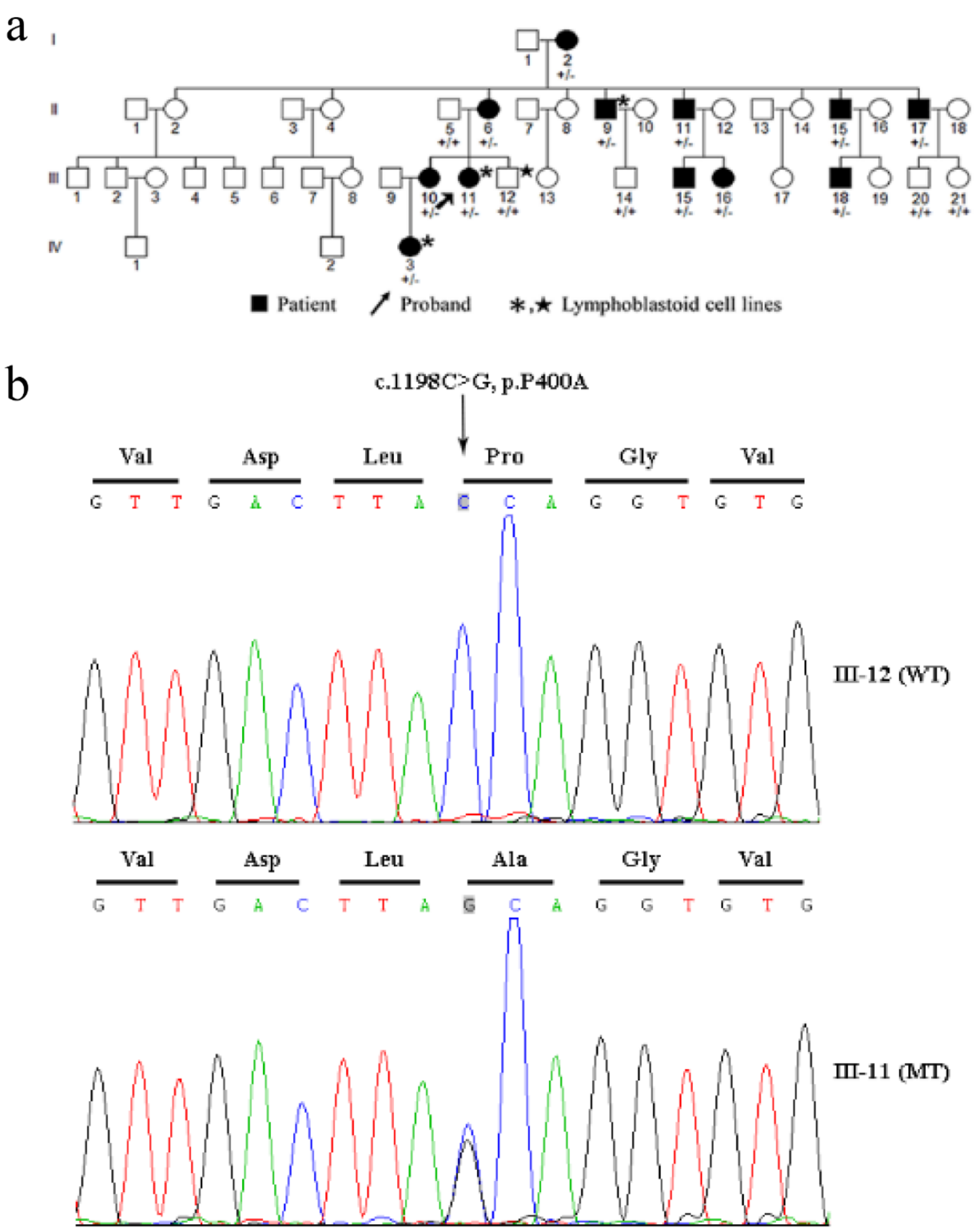


**Supplementary Figure 1. Construction of lymphoblastoid cell lines and identification of cell lines.** (a) Genotype analysis of the Chinese pedigree. Filled symbols: individuals with visual impairment, arrow indicated the proband; +/+: homozygote; +/-: heterozygote; *, Construction of lymphoblastoid cell lines. (b) Partial sequence chromatograms of the *OPA1* gene from lymphoblastoid cell lines of affected individual III-11 and the married-in control subject III-11. Arrow: location of the base changes at position 400.

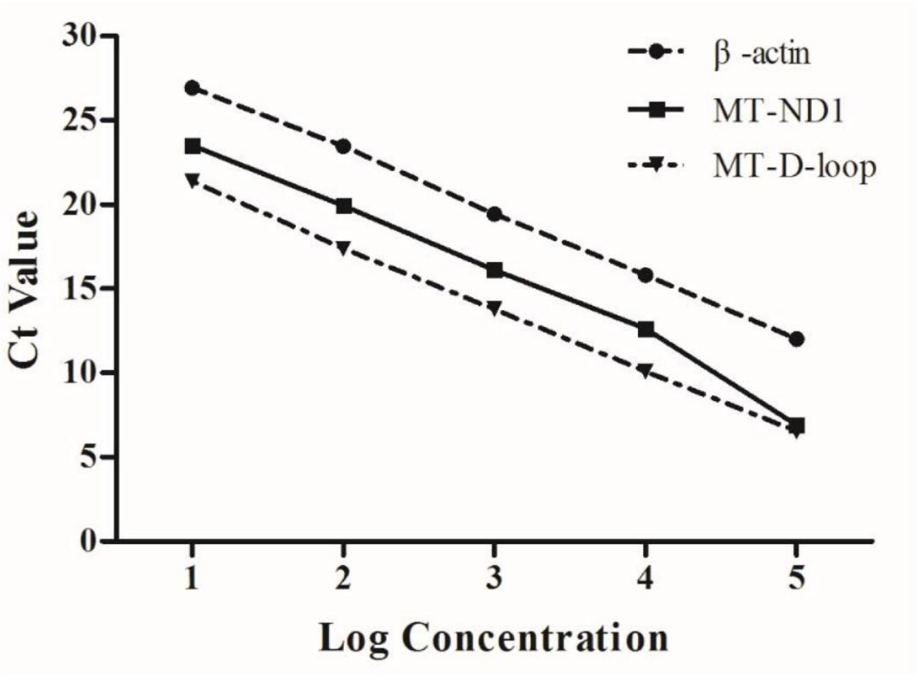


**Supplementary Figure 2. Standard curves of primers in QPCR.** The standard curves were drawn with a ten-fold dilution series ($10^0$-$10^4$) of reference DNA.

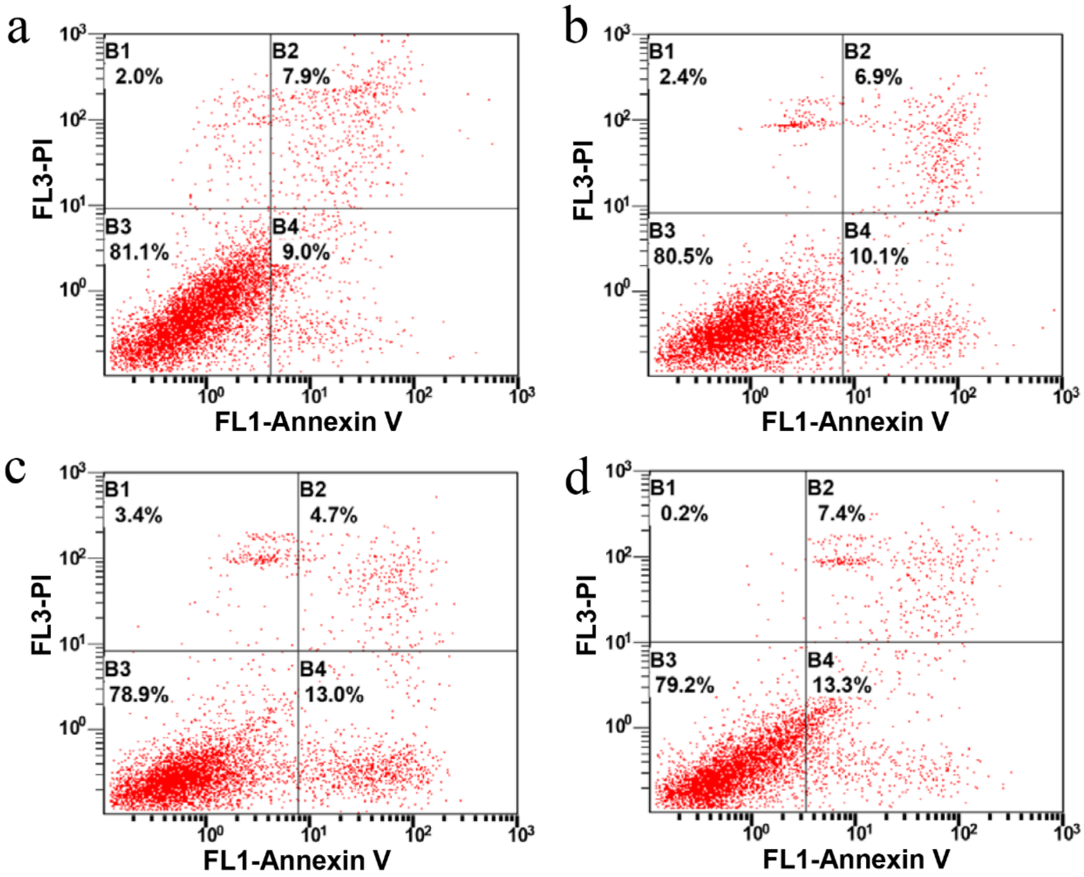


**Supplementary Figure 3. The ratio of apoptotic cells from the third to sixth generation.** Flow cytometric analysis of the third to sixth generation cells stained with both PI and annexin V-FITC to demonstrate the ratio of later stage apoptosis and early necrosis.
